# A Theoretical Analysis of Approaches to Enhance Students’ Grit and Academic Engagement

**DOI:** 10.3389/fpsyg.2022.889509

**Published:** 2022-07-04

**Authors:** Rui Qiao

**Affiliations:** School of Foreign Languages, Xinyang College, Xinyang, China

**Keywords:** EFL students, EFL teacher, academic engagement, positive psychology, grit, positive psychological traits

## Abstract

The role of teachers in developing positive traits in EFL students has been widely endorsed in the literature. Two such traits that have pivotal roles in language teaching and learning are grit and academic engagement. Despite the proliferation of correlational studies on these constructs, theoretical and systematic review studies on the role of EFL teachers’ approaches in strengthening and enhancing these variables are scant. To fill this wide gap, the present study aimed to review the theoretical and empirical underpinnings of students’ grit and engagement and their teachability. In so doing, the definitions, conceptualizations, and dimensions of the two variables were presented. Moreover, a number of practical teaching approaches were suggested to EFL teachers in various contexts. Finally, implications, research gaps, and future directions of this research strand are provided to enhance EFL stakeholders’ knowledge of teachability of grit, engagement, and many other positive psychological traits.

## Introduction

Due to its multi-layered nature and close association with numerous psycho-emotional variables, second/foreign language education has been widely seen as a demanding field (Mercer, 2020). To survive and succeed in such a complicated process, both students and teachers need to deal with an array of factors related to personality, cultural variations, context, psycho-emotional states, and linguistic issues (King and Ng, 2018). Although all these factors are crucial for a successful L2 education, psycho-emotional aspects seem to play a more significant role in learning that was largely ignored in constructivism (Sikma, 2021). In line with this shift of attention from linguistic and cognitive domains to emotions, a new trend in educational psychology, called positive psychology (PP), has gained momentum in the past decades (MacIntyre and Mercer, 2014). Rather than lingering on negative variables like stress, anxiety, boredom, apprehension, and tension, PP proponents argued for the power and influence of positive emotions in having a happier life and a fruitful career (Seligman, 2011; MacIntyre et al., 2019; Gregersen and MacIntyre, 2021). In so doing, different scholarly works have been conducted on the role of various positive emotions/traits in academic success such as credibility, stroke, success (Pishghadam et al., 2021), enjoyment, love, well-being, resilience, self-regulation (Wang et al., 2021), positive mindset (Jin et al., 2020), interpersonal communication skills (Zhang and Zhang, 2020; Xie and Derakhshan, 2021) and many more.

Two other constructs that play a significant role in L2 education, which involves many challenges for both teachers and students, are academic grit and engagement. They are pivotal because lack of toughness in the face of setbacks and disengagement can question many educational attempts. Grit is a non-cognitive spiritual construct that has a close relationship with intrinsic motivation (Ryan and Deci, 2000; Duckworth and Quinn, 2009). It is a disposition that provides energy for the person to use when striving to meet objectives and leads to individual growth (Duckworth, 2016; Fleetwood, 2019). The construct of grit has been identified to cause academic success, interest, and efficiency in both general education and L2 education (Keegan, 2017). Moreover, this positive psychological trait has been approved beneficial in psychology, medicine, and professional performance (Sudina et al., 2021; Yuan, 2022). Owing to its prominent role in PP and SLA research, several correlational studies, in the past decade, have been carried out in different contexts showing that grit can predict students’ involvement and academic performance (Hodge et al., 2018), wellbeing (Jin and Kim, 2017), burnout (Moen and Olsen, 2020), academic success (Strayhorn, 2014; Reed and Jeremiah, 2017), resilience (Shakir et al., 2020), achievement (Steinmayr et al., 2018) and so forth.

Another important area that grit can influence is the academic engagement of the learners in the classroom (Lee, 2020). Engagement concerns students’ degree of involvement in classroom activities that is affected by various personal, contextual, and cultural factors (Guilloteaux, 2016). A large body of research has indicated that affectivity and positive psychological traits, introduced by PP, can influence EFL students’ level of engagement as well (Wilkinson and Kaukko, 2020; Gao, 2021; Zhang, 2021). In other words, when EFL students experience a positive feeling in the process of L2 learning, they are more likely to get engaged in classroom activities/tasks and cope with inherent complexities and challenges of L2 education.

Despite the existence of various correlational studies in this area, practical ways of teaching grit to EFL students *via* appropriate classroom techniques and approaches have been widely ignored in SLA research and practice (Yuan, 2022). Additionally, the main scholarly concern in this area has been identifying the connections of grit and engagement with numerous psycho-emotional factors without paying enough attention to the ways these emotions can be developed *via* practical techniques. Against this lacuna, this review article was a bid to present the theoretical and practical underpinnings of two concepts of grit and engagement in L2 education and presents some practical approaches to teach these constructs in the classroom.

## Background

### The Definitions and Conceptualizations of Grit

As a pivotal element of language education (Duckworth et al., 2007), grit has been defined as a non-cognitive trait that allows people to work hard and follow their long-term desires and goals (Duckworth and Quinn, 2009; Bashant, 2014; Christopoulou et al., 2018). It is rooted in the passion and perseverance that an individual indicates when he/she tries hard to fulfill pre-specified long-term goals despite the existing setbacks (Duckworth and Quinn, 2009). Moreover, Credé et al. (2017) conceptualized grit as a construct equal to conscientiousness, while Duckworth and Quinn (2009) considered it a personality trait beyond motivation in the sense that grit is rather constant across contexts. In contrast to motivation which is dynamic and context-bound, grit hardly changes and focuses on long-term mental strengths, patience, resilience, and determination rather than being an immediately attainable goal (Reed et al., 2013). Grit has been claimed to include two key components of (1) *perseverance in effort* and (2) *persistence of interests* (Duckworth and Quinn, 2009). The former refers to one’s attempt and hunt for achieving his/her goals regardless of encountering setbacks, while the latter points to the consistency and sustainability of one’s passion for obtaining a goal (Duckworth and Quinn, 2009; Duckworth et al., 2010; Xue, 2021).

### The Significance of Grit in Education

In both mainstream education and L2 education, the concept of grit plays an essential role (Keegan, 2017). This is also observable in the current movement in education worldwide to focus the attention on developing students’ competency and skills rather than sticking to trivial information and performances (Horn, 2013). Now, teachers and practitioners are expected to make efforts to inspire and develop students’ practical skills and perseverance to work strenuously to achieve their long-term goals (Taşpinar and Külekçi, 2018). As pinpointed by Duckworth et al. (2010), the purpose of education must be teaching students competence, resilience, and being gritty despite the challenges that they face during the process of learning. Grit is significant in education in that its integration into educational programs can bring about academic success and various similar desired outcomes (Duckworth et al., 2010; Keegan, 2017). As a result, students would become high achievers who are able to deal with the setbacks and obstacles they may face even after their graduation. In other words, they will become permanent problem-solvers (Taşpinar and Külekçi, 2018). Education is not without hard times and annoying complications, hence teachers must take proper approaches to make students tough and buoyant in the face of such challenges creating a sense of determination and strength that ultimately produce high academic performance (Keegan, 2017). Additionally, grit-development orientation in education can bring about favorable outcomes like continuous energy, motivation, self-control, self-discipline, and perseverance in learners as well (Duckworth and Seligman, 2005; Duckworth and Gross, 2014).

### Grit and Second/Foreign Language Research

The construct of grit as a new variable in L2 research has recently gained a growing surge of scholarly attention in various contexts (Yuan, 2022). It has been investigated in relation to various psycho-emotional factors and positive emotions. The results of such studies have signified that grit has a strong correlation with students’ enjoyment, academic success, wellbeing, resilience, achievement, commitment, interest, enthusiasm, academic competence, satisfaction, and scholastic performance (Strayhorn, 2014; Jin and Kim, 2017; Keegan, 2017; Reed and Jeremiah, 2017; Hodge et al., 2018; Steinmayr et al., 2018; Moen and Olsen, 2020; Shakir et al., 2020; Yang, 2021, among other; Wang et al., 2022). Despite the proliferation of correlational research in this line of inquiry, running practical explorations on the teachability and development of grit in EFL contexts is scant. Moreover, the role of teachers as the most important stakeholders who are responsible to ignite and incur learning and developing students’ grit *via* proper approaches is yet to be empirically scrutinized in different EFL/ESL contexts. These gaps indicate that this strand of research is still in its initial steps and deserves more scholarly attention among L2 researchers.

### Students’ Engagement: Definitions and Dimensions

As PP gained momentum in educational psychology, teachers realized the need for paying more heed to students’ academic engagement as a critical factor in generating success in academia (Zhang, 2021). According to Skinner and Pitzer (2012), the concept of student engagement pertains to the degree and duration of students’ involvement in the classroom activities provided by their teacher. It is an optimal goal in language learning that paves the way for many other human competencies (Sinatra et al., 2015). The construct has been perceived as a clear indication of motivation that affords energy for learners’ academic effort and success (Phillips, 2015).

**FIGURE 1:**
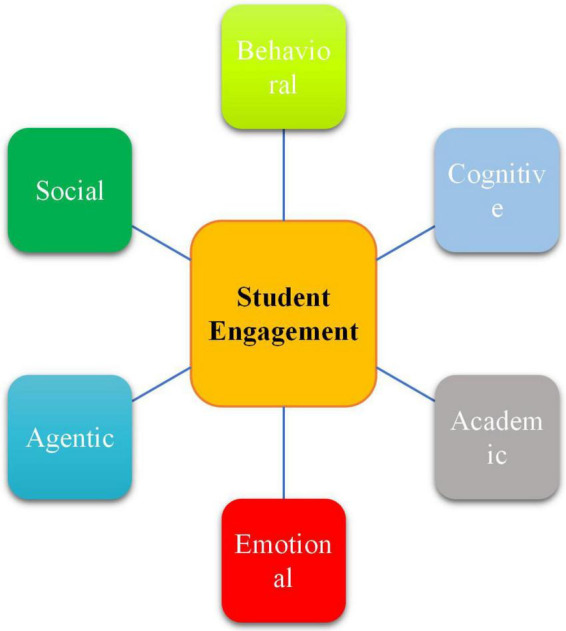
The dimensions of student engagement.

As stated by DeVito (2016), this dynamic construct has different dimensions such as behavioral, emotional, cognitive, agentic, academic, motivational, and social dimensions. Such dimensions are by no means mutually exclusive but are in many part-to-part, part-to-whole, and whole-to-part interactions that cause overall classroom engagement (Symonds et al., 2021). In a similar vein, Reschly and Christenson (2012) provided the descriptions of different dimensions of engagement (Figure 1) as following:

• *Behavioral engagement*: refers to students’ active classroom participation through the given tasks/activities.
• *Cognitive engagement:* has to do with students’ psychological investments during their learning process using complicated strategies during task completion.
• *Emotional engagement:* concerns students’ internal emotions, inner states, and their affective reactions to learning.
• *Academic engagement:* is about different psychological and behavioral attempts that students make to master an academic skill.
• *Agentic engagement:* pertains to students’ degree of agency and involvement in both learning and teaching processes.
• *Social engagement:* refers to students’ degree of involvement in social-based tasks and activities whose goal is to inspire their problem-solving and social interaction abilities.

### Research on EFL Students’ Engagement

The concept of student engagement as one of the most desirable positive traits boomed by PP movement in both general education and L2 education has witnessed a growing body of research in different contexts. The obtained research evidence demonstrated that engagement is positively correlated with many psycho-emotional factors like motivation, resilience, ambiguity tolerance, agency, effective learning, persistence, retention, willingness to communicate (WTC), and learning perception (Wang and Guan, 2020; Zhang, 2021). In addition to these positive academic outcomes, it has been identified that student engagement can exponentially assist in shaping students’ socialization and psychological wellbeing and generating life satisfaction. Likewise, based on the existing literature in this domain, engagement has a robust relationship with many other positive constructs common in PP including joy, passion, buoyancy, stroke, interpersonal communication skills, care, hope, closeness, connectedness, and so on that deserve additional research. The missing point in researching this variable in L2 education is that the current body of knowledge is limited to correlational studies focusing on the statistical relationships that engagemen has with other personal-emotional variables. Moreover, running practical studies highlighting the role of particular teaching approaches in developing this emotion in students has been widely ignored, to date. This is where the present review article can add to the body of knowledge considering the role of teachers’ pedagogical practices in shaping and reshaping their students’ positive psychological traits, which has been largely confined to one-shot correlational studies.

## Approaches to Enhance and Strengthen Students’ Grit and Academic Engagement

Teachers are expected to devise novel teaching approaches and techniques when it comes to developing students’ emotions and inner feelings. The available literature on grit and academic engagement lack sufficient practical teaching approaches to develop these two positive traits in EFL/ESL contexts. Nevertheless, there are some useful ways by which EFL teachers can enhance students’ grit and engagement. First, both constructs are changeable in case teachers provide a positive classroom culture/climate in which the focus is shifted toward character development (Dean, 2014). In such a setting, teachers can teach students important skills and involve them in social and school issues and decisions. Second, EFL teachers can design character-building courses in which they make attempts to strengthen students’ positive psychological traits like grit and engagement aside from instructional issues. Third, teachers can establish a trusting and caring environment in which the interactions and interventions are more significant in comparison to strategies (Pappano, 2013). Fourth, students’ grit and engagement can be enhanced through a wise juxtaposition with other positive constructs such as stroke, rapport, social support, and interpersonal communication skills (credibility, clarity, confirmation, and immediacy). This is well-substantiated by a recent study conducted by Yuan (2022) who examined the role of teacher stroke and rapport with students in developing Chinese EFL students’ grit. Despite these insightful approaches, depending on teachers’ experience and context, many more approaches can be proposed to enhance EFL students’ grit and engagement that is obtainable by running complementary studies. In other words, it is significant to move beyond simple correlations between positive constructs in L2 education and, instead, securitize the practical aspects of developing and generating positive outcomes in students through the lens of EFL teachers.

## Concluding Remarks

In this review article, it was argued that EFL students’ grit and academic engagement are teachable and likely to develop *via* teachers’ appropriate teaching approaches such as character-building programs and designing courses in which these two emotions are dealt with in connection with other PP constructs. Hence, it is opined that many students’ emotions can be taught and improved through professional and judiciously done approaches which, in turn, generate many other optimal outcomes in academia. Yet, these desired consequences are only achievable in case teachers have required expertise in developing and dealing with students’ emotions. In light of these concerns, the present study can be useful for EFL teachers in that they can identify some practical approaches to strengthen their students’ grit and classroom engagement. They can also use the ideas mentioned in the article to start developing and testing their own techniques and approaches to develop students’ positive emotions. EFL teacher educators, too, can use this review to propose training courses to novice and experienced EFL/ESL teachers focusing on preparing them to deal with students’ positive emotions *via* proper teaching techniques and approaches taught in the professional development programs. Moreover, SLA researchers may find this article beneficial in that they can bridge the existing gaps in this area. More specifically, they can take advantage of qualitative, mixed-methods, longitudinal, and experimental research designs to explore students’ grit and engagement which offer more vivid images. Future researchers can use the mentioned designs to capture the constructs of grit and academic engagement more comprehensively in contrast to one-shot, correlational designs dominant, to date. Future investigations are recommended to scrutinize the developmental trajectories of these two constructs in time-series research designs as they are dynamic and subject to change across time and context. The impact of training courses and special teaching approaches on students’ positive psychological traits is also an interesting line of research for future scholars. More particularly, future studies can be carried out to examine the efficacy of specific approaches to increase students’ grit and engagement to be used in other contexts as well. Finally, the role of contextual and socio-cultural factors in the development of students’ grit and engagement has been limitedly explored and this has provided a fresh topic for further research. Although this review study is promising in that it signifies the criticality and insightfulness of studying teachers’ role in enhancing grit and academic engagement, its scope is limited to these two constructs. This line of research can be complemented by demystifying the teachability of many other positive emotions in education.

## Author Contributions

The author confirms being the sole contributor of this work and has approved it for publication.

## Conflict of Interest

The author declares that the research was conducted in the absence of any commercial or financial relationships that could be construed as a potential conflict of interest.

## Publisher’s Note

All claims expressed in this article are solely those of the authors and do not necessarily represent those of their affiliated organizations, or those of the publisher, the editors and the reviewers. Any product that may be evaluated in this article, or claim that may be made by its manufacturer, is not guaranteed or endorsed by the publisher.
